# Time‐resolved mapping of myocardial stiffness using 2D multifrequency spiral MR elastography with and without external vibration

**DOI:** 10.1002/mrm.70007

**Published:** 2025-07-28

**Authors:** Matthias S. Anders, Carsten Warmuth, Tom Meyer, Helge Herthum, Mehrgan Shahryari, Jakob Schattenfroh, Corona Metz, Jan Bieling, Josef Pfeuffer, Simon Veldhoen, Jeanette Schulz‐Menger, Tobias Schaeffter, Jing Guo, Heiko Tzschätzsch, Ingolf Sack

**Affiliations:** ^1^ Division of Pediatric Radiology Charité – Universitätsmedizin Berlin, Corporate Member of Freie Universität Berlin and Humboldt‐Universität zu Berlin Berlin Germany; ^2^ Department of Radiology Charité – Universitätsmedizin Berlin, Corporate Member of Freie Universität Berlin and Humboldt‐Universität zu Berlin Berlin Germany; ^3^ Berlin Center for Advanced Neuroimaging (BCAN), Berlin, Germany, Corporate Member of Freie Universität Berlin Berlin Institute of Health and Humboldt‐Universität zu Berlin Berlin Germany; ^4^ Application Development Siemens Healthineers AG Erlangen Germany; ^5^ Charité – Universitätsmedizin Berlin, Corporate Member of Freie Universität Berlin and Humboldt‐Universität zu Berlin Berlin Germany; ^6^ Working Group On CMR, Experimental and Clinical Research Center, a cooperation between Charité ‐ Universitätsmedizin Berlin and Max Delbrück Center for Molecular Medicine in the Helmholtz Association Berlin Germany; ^7^ DZHK (German Center for Cardiovascular Research), partner site Berlin Berlin Germany; ^8^ Department of Cardiology and Nephrology HELIOS Hospital Berlin‐Buch Berlin Germany; ^9^ Physikalisch‐Technische Bundesanstalt (PTB), Braunschweig and Berlin Berlin Germany; ^10^ Department of Medical Engineering Technische Universität Berlin, Einstein Center Digital Future Berlin Germany; ^11^ Institute of Medical Informatics Charité – Universitätsmedizin Berlin, Corporate Member of Freie Universität Berlin and Humboldt‐Universität zu Berlin Berlin Germany

**Keywords:** endogenous harmonic motion, heart, magnetic resonance elastography, multifrequency cardiac MRE, spiral readout, stiffness

## Abstract

**Purpose:**

There is a clinical need for stiffness mapping of the heart; however, current cardiac magnetic resonance elastography (cMRE) has limited spatiotemporal resolution. Therefore, we developed 2D spiral multifrequency MRE of the human heart and conducted a study to analyze the consistency and reproducibility of motion‐encoding and stiffness mapping with and without external vibration.

**Methods:**

Eleven healthy volunteers were studied using single‐slice gradient‐echo spiral cMRE with cardiac triggering and encoding of harmonic shear wave fields at 70, 80, and 90 Hz frequency generated by either external drivers or endogenous heart motion. Tissue displacement was monitored synchronized to the cardiac cycle, and frequency‐resolved shear wave speed (SWS) maps were reconstructed as a proxy for left ventricular (LV) stiffness variations. After several days, all subjects underwent repeat scanning for reproducibility analysis based on intraclass correlation coefficients (ICCs).

**Results:**

cMRE with external vibration showed LV SWS to be highest in end‐systole (ES) (2.17 ± 0.23 m/s), followed by diastole (DIA) (1.94 ± 0.15 m/s) and isovolumetric contraction (IVC) (1.78 ± 0.18 m/s). ICCs decreased with distance from the R‐wave from excellent (0.93) in IVC to moderate (0.68) in ES. Without external vibration, sufficient LV harmonic displacement amplitudes permitted SWS reconstruction, resulting in similar SWS, but lower ICC values than with external vibration in IVC and ES.

**Conclusions:**

Multifrequency cMRE offers high spatiotemporal resolution and reproducibility with external vibration. In addition, the technique allows the encoding of endogenous shear waves during cardiac phases with pronounced wall motion.

## 1 INTRODUCTION

The heart propels blood through the cardiovascular circuit by mechanical forces generated by the cardiac walls during contraction.^1^ Myocardial contraction is driven by the collective binding of myosin to actin filaments and is associated with global cardiac tissue stiffening.^2^ Elastography measures the stiffness of biological tissues using medical imaging techniques such as MRI and ultrasound.^3^ In the heart, it can be used to directly assess myocardial function.^4^, ^5^, ^6^, ^7^

However, cardiac elastography is challenging because of rapid contraction motion, slow breathing motion, complex cardiac geometry, passive and active stiffening, and muscle fiber anisotropy.^8^ Consequently, researchers have developed and tested a wide variety of cardiac elastography techniques ranging from time‐harmonic tissue stimulation in MRI^9^, ^10^, ^11^, ^12^, ^13^ and ultrasound^14^ to transient approaches^5^, ^15^, ^16^ and utilization of endogenous myocardial deformation.^7^, ^17^, ^18^, ^19^

MR elastography (MRE) has been established for many clinical diagnostic applications, including detection of liver fibrosis,^20^, ^21^ tumor characterization,^22^, ^23^ and therapy monitoring.^24^ Cardiac MRE (cMRE) has revealed sex differences in age‐related myocardial stiffening,^25^ abnormal left ventricular (LV) stiffness in patients with amyloidosis,^26^ diastolic dysfunction,^12^ and LV hypertrophy.^9^ Larger clinical trials and translation of cMRE into clinical practice are pending because of various challenges such as lengthy imaging protocols, the need for additional hardware for shear wave excitation, and limited spatiotemporal resolution of quantitative stiffness maps. We hypothesize that quantitative cMRE with high spatiotemporal resolution may facilitate the investigation of stiffness‐related cardiac pathophysiology.

We present multifrequency gradient‐echo spiral cMRE with cardiac triggering and 40 Hz temporal resolution of serial stiffness maps over the cardiac cycle. Similar to a recent multifrequency cMRE study by Castelein et al.,^13^ shear wave fields are encoded at three frequencies from 70 to 90 Hz. To achieve a high frame rate, multislice wave field acquisition is sacrificed in favor of single‐slice wave field acquisition. This strategy attempts to combine the advantages of rapid ultrasound elastography^27^ with the capabilities of MRI to encode full Cartesian wave fields. In addition, the high frame rate of our cMRE technique is exploited to encode intrinsically stimulated shear waves at the same harmonic frequencies as generated by external actuators. All subjects were re‐examined after several days to investigate the reproducibility of cMRE with and without external vibration.

If feasible, quantification of LV stiffness in 2D, yet with high spatiotemporal resolution, may directly reveal impaired mechanical function associated with heart failure in a range of conditions including diastolic dysfunction. The data presented are intended to encourage further efforts to improve cMRE with or without externally induced vibration, toward stiffness mapping as a biomarker in cardiac MRI.

## 2 METHODS

Eleven volunteers (male; mean age, 31 ± 7 years) without any symptoms or a history of cardiac disease underwent two cMRE examinations separated by 7 days. The study was approved by our institutional review board (IRB), and all volunteers gave written informed consent before the examinations.

**FIGURE 1:**
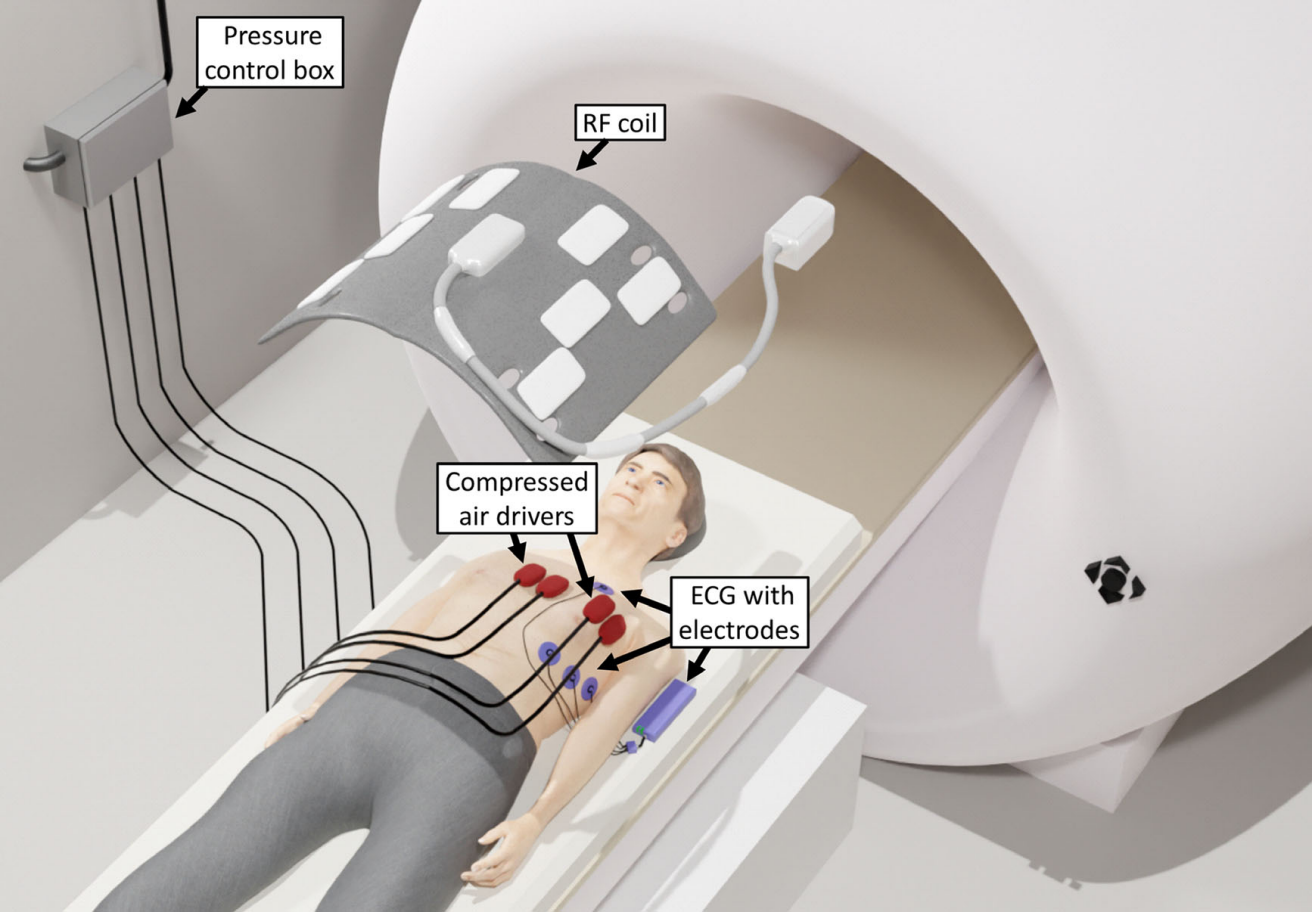
Cardiac MR elastography (MRE) setup. Four air actuators, two near the sternum and two near the left pectoral region, are placed on the chest (red) and connected by plastic tubing to the pressure control box, which generates periodic compressed‐air pulses at the desired MRE frequency. MRE image acquisition with the surface coil is electrocardiogram (ECG)‐triggered in synchrony with the phase of the externally induced vibrations.

**FIGURE 2:**
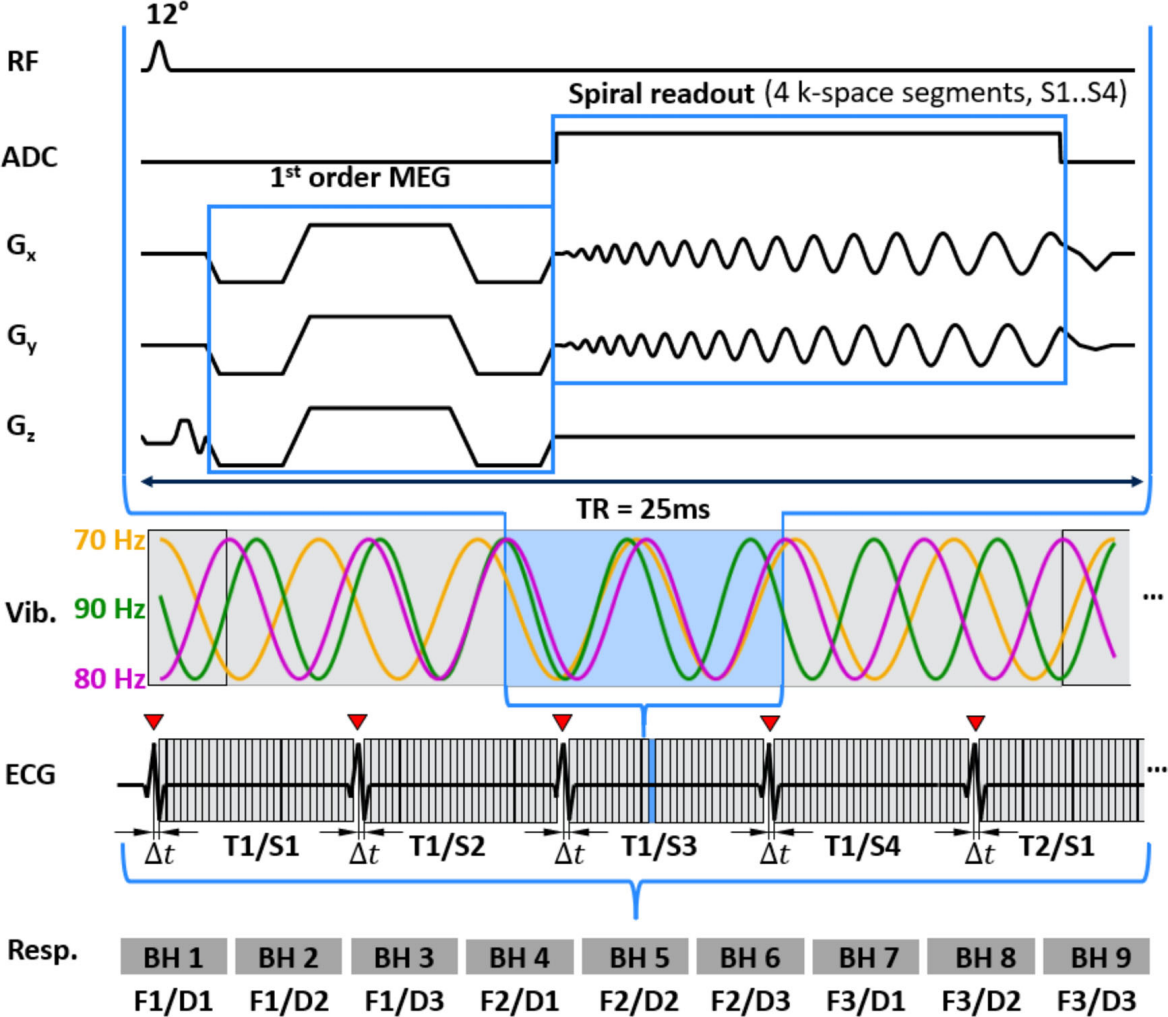
Timing diagram of the cardiac gradient‐echo spiral MR elastography (MRE) sequence. The imaging sequence over one TR is shown on the top. After detection of an R‐wave from the electrocardiogram (ECG) (red triangles), the sequence is synchronized by a short delay Δt to ensure encoding of one of six wave dynamics (time points T1…T6 during a wave period). One of four spiral k‐space segments (S1…S4) is acquired per TR with first segments S1 at first wave dynamic T1 in the R–R series of, for example, 30 images, followed by segments S2 of the same wave dynamic T1. In this way, the k‐spaces of all 30 images within an R–R interval are filled up with four spiral segments before the next wave dynamic, T2, is acquired. All six wave dynamics, T1…T6, are sampled within a 23‐s breath‐hold. This sequence with vibration frequency F1 and motion‐encoding gradients (MEG) direction D1 is then repeated for MEG directions D2 and D3 and frequencies F2 and F3, requiring a total of nine breathholds. BH, breathhold; D1…D3, directions of space diagonals of the MEG according to the imaging frame; F1…F3, external vibration frequency; Resp, respiration state; S1…S4, spiral segments in k‐space; T1…T6, time steps over a wave period; Vib, external vibration.

All subjects were examined in a 3‐T MRI scanner (MAGNETOM Lumina, Siemens Healthineers) using a 12‐channel body coil. Acoustic chest vibrations at frequencies of 70, 80, and 90 Hz were generated by four custom‐made compressed air drivers as shown in Figures 1 and 2. Two drivers were attached to the sternum and two to the left pectoral region using Velcro stripes. A 4‐lead electrocardiogram (ECG) was used to synchronize sequence timing with the cardiac cycle.

### 2.1 Data Acquisition

**FIGURE 3:**
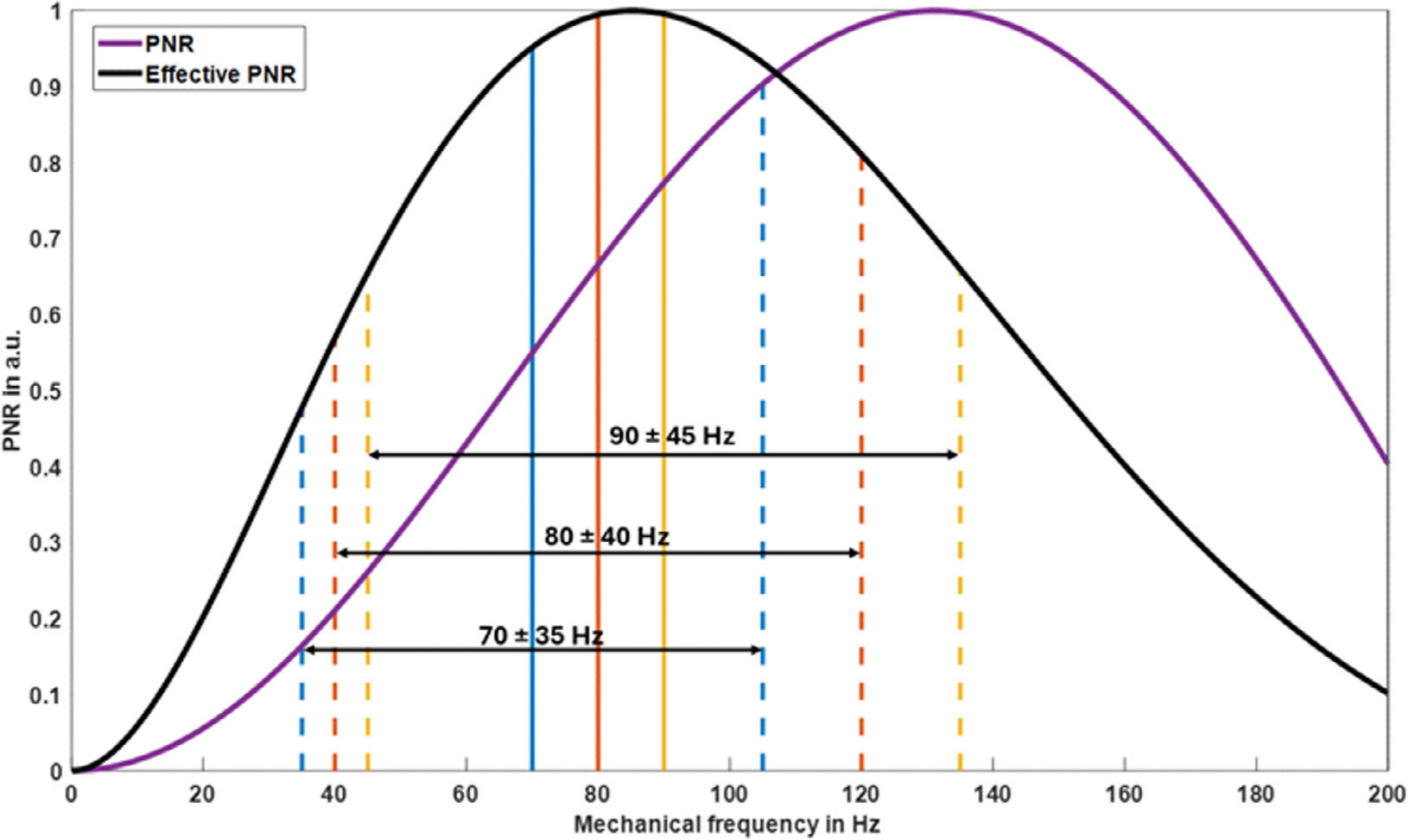
Normalized phase‐to‐noise ratio (PNR) based on the encoding efficiency of a rectangular motion‐encoding gradients (MEG) with balanced first momentum and 8.75 ms duration, plotted over a spectrum of mechanical frequencies. Shown are PNR for equal mechanical vibration amplitudes over the entire spectrum (purple curve) and effective PNR considering fractional encoding of exponentially damped higher frequencies according to the Voigt model as described in Rump et al.^53^ (black curve, shear modulus *μ* = 6 kPa, viscosity *η* = 4.5 Pa‐s, values correspond to 2.4 m/s shear wave speed and medium tissue viscosity^54^). Vertical lines delineate the encoding efficiencies of precisely known externally induced vibrations at 70, 80, and 90 Hz (solid lines) and the corresponding encoded frequency bandwidths (dashed lines), defined by the sequence timing for each frequency.

Before data acquisition, vibrations were generated for 1 s of calibration time to ensure an oscillatory steady state. In addition, an R–R interval was invested to establish a steady state of proton magnetization by applying a sequence of RF pulses without data acquisition. The sequence design is shown in Figure 2. Spatial encoding was accomplished using a multishot gradient‐echo spiral sequence with dual density readout. Motion‐encoding gradients (MEG) with nulled first‐order moments were used to sensitize the sequence to motion. The k‐space was sequentially acquired by four spiral interleaves with full sampling near the center of k‐space and twofold undersampling in outer regions.^28^ A single image slice was acquired in a short‐axis view placed within the mid LV cavity. The following acquisition parameters were used: TR = 25 ms, TE = 10 ms, flip angle (FA) 12°, FOV 192 × 192 mm^2^, voxel size 2.0 × 2.0 × 8.0 mm^3^, and encoding by three MEGs of amplitude 34 mT/m, each simultaneously deployed along the Cartesian axes (x, y, z) of the scanner coordinate system resulting in an overall gradient strength of $\sqrt{3}$ × 34 mT/m. Based on the 4‐point tetrahedral encoding scheme described by Guenthner et al.,^29^ three of the four wavefield components were acquired to use the increased encoding efficiency and shorten scan duration by consecutively switching MEG directions from +x, +y, +z to +x, −y, +z and +x, +y, and −z. Notably, these encoded space diagonals do not form an orthogonal system, but are aligned along three axes of a tetrahedral system. Therefore, they potentially cause artifacts when inversion algorithms assume Cartesian field components, as is the case with the curl operator. Furthermore, the contribution of imaging gradients should be considered, which however, was negligible in our sequence, with only 0.17% of MEG sensitivity generated by the flow‐compensated slice selection gradient. The MEG duration of 8.75 ms resulted in encoding efficiencies of 51.7, 62.7, and 72.7 rad/mm for $f_{\text{mech}}$ = 70, 80, and 90 Hz mechanical frequency, respectively. Six wave phases were acquired with equal intervals over $1/f_{\text{mech}}$ providing a spectral sensitivity of $f_{\text{mech}}\pm 0.5f_{\text{mech}}$, as shown in Figure 3 with maximum effective phase‐to‐noise ratio (PNR) near the center frequency of 80 Hz. Data acquisition was initiated at the R‐wave including a short delay of at most one vibration period for synchronizing the sequence to the desired wave phase. Twenty‐five to 35 TRs of individual images were acquired consecutively, depending on the subject's heart rate, to ensure that the acquisition of a k‐space segment in an image sequence did not exceed one R–R interval. Data acquisition for three MEG‐combinations and three frequencies was performed in a total of nine breathholds of approximately 23 s at end‐expiration. Before MRE, single‐slice, cardiac‐phase resolved field maps were acquired every 47 ms (with a total of 14–19 field maps, depending on the subject's heart rate) using two consecutive scans with different TE (4.9 and 6.5 ms) to correct for B_0_ inhomogeneities. Therefore, 10 dual‐density spiral interleaves were acquired with full sampling near the center of k‐space and twofold undersampling in outer regions.^28^ Similar to cMRE scans, field maps were acquired at end‐expiration within 21 heartbeats, including one dummy cycle. Further parameters for field mapping: FA 20°, FOV 384 × 384 mm^2^, and voxel size 2.0 × 2.0 × 8.0 mm^3^. One full cMRE examination, including short‐axis slice localization, field mapping, motion data acquisition with and without external vibration, and respiratory pauses, took approximately 12 min.

### 2.2 Postprocessing

MRI images were reconstructed online using Tan's algorithm^30^ and exported for further processing. Before inversion, the complex MRI images were denoised by singular value decomposition (SVD).^31^ Therefore, each series of wave images over all cardiac phases and for any specific MEG direction‐frequency combination was first cropped to the heart region and then restructured to a 2D spatiotemporal array as explained in the Appendix. Animations of original and SVD‐treated phase images with and without external vibration are shown in Video S1 indicating that no spurious wave motion is generated. The phases of the SVD‐filtered complex, heart‐region specific MRI data were unwrapped using the MATLAB code for n‐dimensional phase unwrapping provided in Dittman et al.^32^ The temporal Fourier transformation (t‐FT) was applied in each voxel along the six time steps over one wave cycle to generate complex‐valued wave images at fundamental frequency and to suppress background phase inhomogeneities. Wavenumber (k‐) based multi‐inversion (k‐MDEV) was applied to reconstruct stiffness maps in terms of shear wave speed (SWS) (in m/s), as detailed in Tzschätzsch et al.,^33^ using the default parameter settings for abdominal/pelvic multifrequency MRE provided on https://bioqic‐apps.charite.de.^34^ k‐MDEV with the default parameter setting for abdominal and pelvic organs was used to ensure the diagnostic power of our method also outside the heart. The encoded tetrahedral components of the wave field were directly processed without rotation into a Cartesian coordinate system as k‐MDEV inversion does not require orthogonal components. However, as cropped wave images were processed, a zero padding of 30 voxels was applied around the edges of each wave image before the spatiotemporal filtering implemented into the k‐MEDV inversion.^35^ After filtering, the zero pads were deleted and images were further processed according to the standard k‐MDEV pipeline to ensure comparability to diagnostic MRE.^36^ For consistency, the same inversion procedure was applied to cMRE wave images with and without external vibrations. However, because $f_{\text{mech}}$ without vibration is unknown, we report endogenous cMRE values based on the dominating frequency range of 70 to 90 Hz as analyzed in Figure 3. Henceforth, endogenous SWS will be taken as apparent SWS related to the three dominant harmonic frequencies, 70, 80, and 90 Hz, to which the timing of our cMRE sequence was synchronized.

### 2.3 Spatiotemporal regions of interest

**FIGURE 4:**
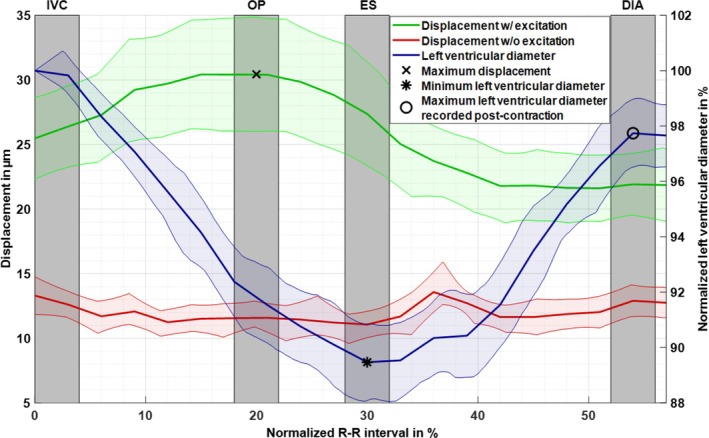
Time evolution of frequency‐ and encoding direction‐averaged harmonic displacement in the left ventricular (LV) myocardium at 70, 80, and 90 Hz induced by external vibration (green) and endogenous harmonics without external vibration (red) over normalized R–R interval. For comparison, the change in LV diameter is shown in blue. Group means are represented by solid lines and 95% confidence intervals by colored shaded regions. Cardiac phases of interest are demarcated in gray. The detected R‐wave peak was defined as the beginning of the isovolumetric contraction phase (IVC), whereas the maximum total displacement amplitude (marked by a cross) defined the center of the optimal phase (OP). The minimum LV diameter (marked by an asterisk) defined the center of the end‐systolic phase (ES). The center of the diastolic phase (DIA) (marked by a circle) was defined by the maximum LV diameter at end‐diastole. All intervals of interest extended over 4% (40 ms) of the normalized R–R range.

Regions of interests (ROIs) in the LV myocardium were manually drawn for each cardiac phase based on the MRE magnitude images averaged over MEG directions and excitation frequencies. Demarcation of cardiac phases for statistical analysis is shown in Figure 4. The beginning of the isovolumetric contraction phase (IVC) was defined by the R‐wave, whereas the maximum total displacement amplitude defined the center of the optimal phase (OP). The minimum LV diameter defined the center of the end‐systolic phase (ES), and the center of diastolic phase (DIA) was defined by the maximum LV diameter at end‐diastole. All intervals of interest extended over 4% (40 ms) of the normalized R–R interval of 1 s.

### 2.4 Statistics

Mean values were calculated by averaging SWS measured within the spatiotemporal ROIs. The paired *t*‐test was used to check for differences in SWS between IVC, ES, and DIA and differences in displacement amplitudes (DAs) between cardiac phases. Repeatability between test and re‐test was assessed using the two‐way mixed‐effects model, absolute agreement, and single‐rater intraclass correlation coefficients (ICCs). ICCs <0.5, 0.5 to 0.75, 0.75 to 0.9, and >0.90 indicated poor, moderate, good, and excellent repeatability,^37^ respectively. A *p*‐value <0.01667 (Bonferroni‐corrected) was considered statistically significant. Statistics were calculated only for phases of the normalized R–R interval where data were available for all volunteers.

## 3 RESULTS

cMRE was successfully applied in all volunteers. The proposed SVD denoising and image cropping before k‐MDEV inversion did not affect SWS values, as demonstrated by consistent group‐mean liver values of 1.6 ± 0.3 m/s obtained by both novel cMRE and established abdominal k‐MDEV inversion^34^ (*p* > 0.05). Figure 4 shows group‐averaged wave amplitudes in the LV myocardium with and without vibration along with LV diameters over the normalized R–R interval. LV diameter was normalized to 100% at the beginning of the IVC phase, followed by cardiac contraction toward minimum values at ES, and subsequent increases during myocardial relaxation in DIA. The center of DIA was defined by the maximum LV diameter after ES. Displacement amplitudes were averaged across all vibration frequencies and motion‐encoding directions for each subject. With vibration, DAs were approximately 25.5 ± 7.6 μm at the beginning of IVC and increased to peak values of 30.4 ± 10.5 μm at 20% of the R–R interval, followed by leveling at 21.8 ± 6.1 μm for the rest of the R–R‐interval. The cardiac phase with the highest extrinsically induced DAs defined the center of optimal phase (OP) for cMRE. Without vibration, averaged DAs were highest at the beginning of IVC with 13.3 ± 3.5 μm and at 36%, after aortic valve closure, with 13.6 ± 3.7 μm. These cardiac phases are characterized by more endogenous motion than other cardiac phases such as OP^5^, ^17^ in which DAs were significantly lower (11.5 ± 3.3 μm, *p* < 0.01). In‐plane shear strains based on the curl operator in the LV myocardium and in the liver are shown in Figure S1 as supplemental material. Therefore, the tetrahedral field components were converted into a orthogonal system. Notably, liver strain near the heart remained constant over the entire R–R interval suggesting consistently encoded vibrations despite potential respiratory artifacts in k‐space sampling.

**FIGURE 5:**
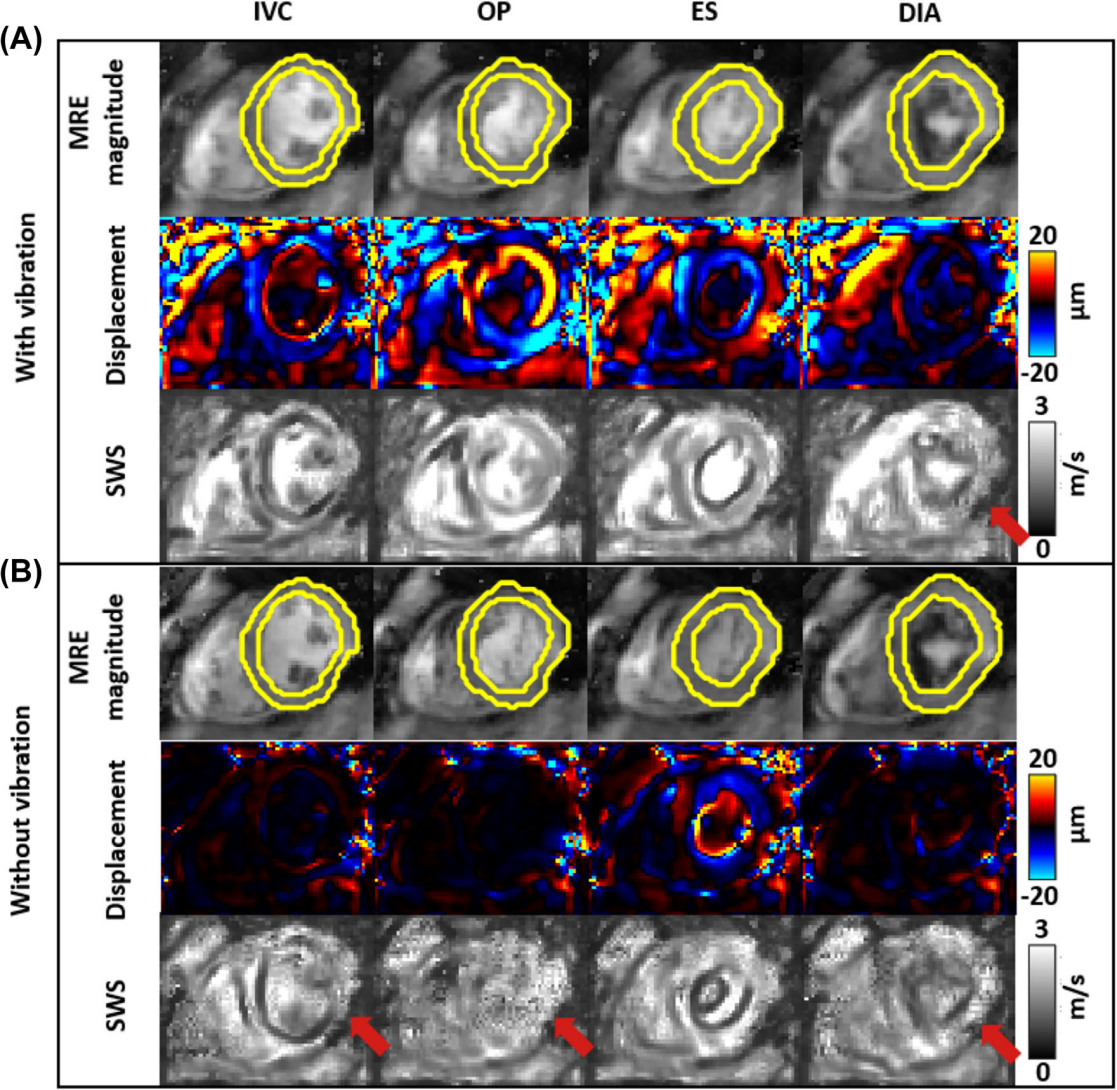
Representative MR elastography (MRE) magnitude images, displacement maps, and shear wave speed (SWS) maps in cardiac phases isovolumetric contraction (IVC), optimal phase (OP), end‐systolic (ES), and diastole (DIA) in a short‐axis view. (A) Maps obtained with vibration. (B) Maps obtained without vibration. In the top rows, MRE magnitude images are shown along with regions of interest (ROIs) placed in the LV myocardium, demarcated by yellow lines. The middle rows show the displacement component encoded by the +x + y + z MEGs at 80 Hz (real parts of the complex wave images). The bottom rows show compounded SWS maps based on three frequencies and three displacement directions. Inversion artifacts because of noise and non‐periodic cardiac motion appear as pixelated noise patterns in SWS maps (red arrows).

Figure 5 shows representative MRE magnitude images, wave images, and SWS maps obtained with and without external vibration in IVC, OP, ES, and DIA. External vibration appeared not to affect the quality of MRE magnitude maps. In all cardiac phases, harmonic displacement amplitudes were higher with vibration than with non‐vibration cMRE, suggesting the importance of sufficiently high vibration amplitudes for imaging anatomical detail without high‐frequency pixel artifacts (pixelation) in SWS maps. However, SWS pixelation was also visible during DIA when external vibration was used, indicating an increasing inconsistency between expected and true cardiac phases with increasing time delay from the ECG‐detected R‐wave. Video S2 shows animated wave images obtained at different frequencies and motion‐encoding directions with regions of confidence demarcating wave‐averaged amplitudes greater than 3.6 μm as suggested in Anders et al.^38^ Figure S2 shows SWS maps with outlines of the regions of confidence while Figure S3 shows corresponding SWS maps at single harmonic frequencies.

**FIGURE 6:**
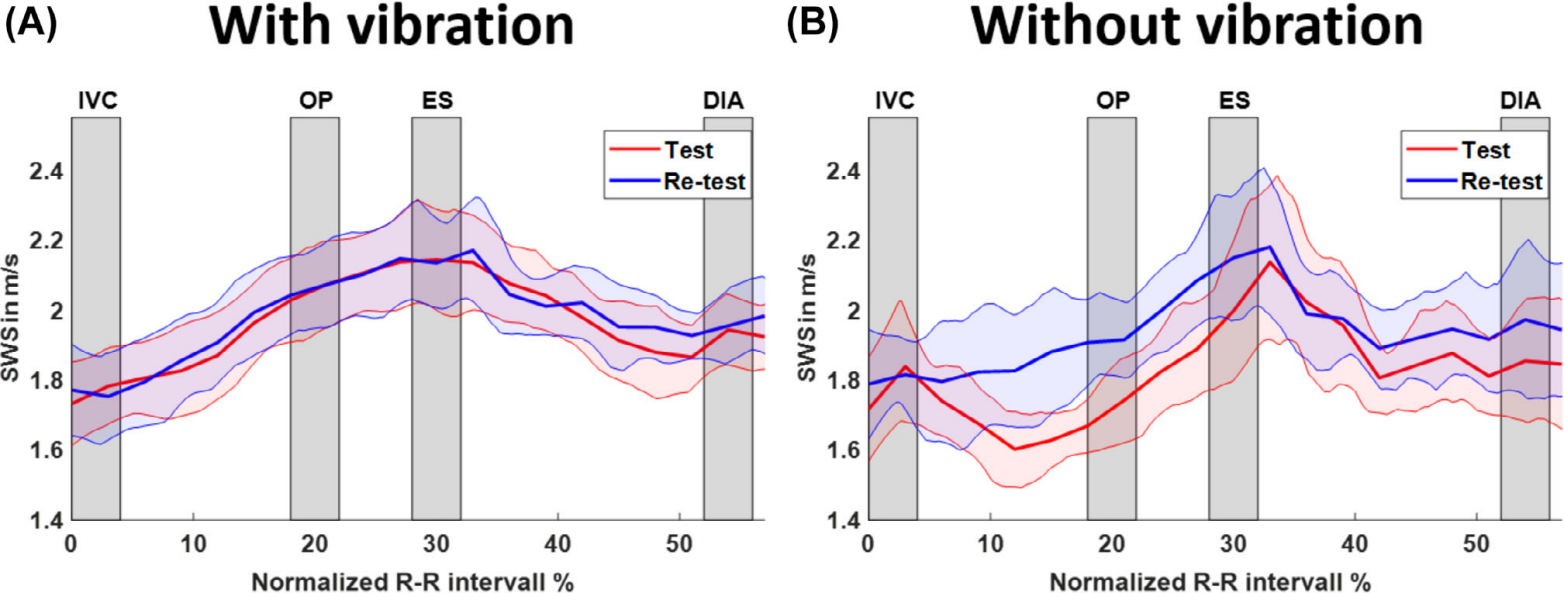
Time evolution of group mean shear wave speed (SWS) within the left ventricle over normalized R–R interval for test (blue) and re‐test (red). 95% confidence intervals are shown as colored shaded areas with cardiac intervals of interest demarcated in gray. (A) SWS obtained with external vibration. (B) SWS obtained from endogenous shear waves without external vibration. Optimal test–re‐test reproducibility across the R–R interval was achieved with external vibration, whereas smaller endogenous harmonic amplitudes (see Figure 4) gave rise to greater SWS variability. DIA, diastole; ES, end‐systole; IVC, isovolumetric contraction; OP, optimal phase.

Figure 6 shows the evolution of composite SWS in the LV over the normalized R–R interval, incorporating all motion‐encoding directions and frequencies. Figure 6A shows that SWS measured with vibration increased from IVC (test: 1.76 ± 0.17 m/s; re‐test: 1.76 ± 0.17 m/s; ICC: 0.93) to OP (test: 2.06 ± 0.20 m/s; re‐test: 2.06 ± 0.20 m/s; ICC: 0.86), peaked at ES (test: 2.06 ± 0.20 m/s; re‐test: 2.06 ± 0.20 m/s; ICC: 0.77), and then declined toward DIA (test: 1.93 ± 0.15 m/s; re‐test: 1.96 ± 0.15 m/s; ICC: 0.66). Figure 6B displays SWS without vibration, showing a similar trend, but less smooth evolution over time. SWS gradually increased from IVC (test: 1.80 ± 0.24 m/s; re‐test: 1.81 ± 0.24 m/s; ICC: 0.72) to OP (test: 1.72 ± 0.17 m/s; re‐test: 1.92 ± 0.17 m/s; ICC: 0.21), peaked at ES (test: 2.00 ± 0.30 m/s; re‐test: 2.16 ± 0.30 m/s; ICC: 0.59), and decreased at DIA (test: 1.85 ± 0.26 m/s; re‐test: 1.96 ± 0.26 m/s; ICC: 0.45). Test and re‐test curves showed greater alignment with vibration. The decreasing ICC values over the cardiac cycle with vibration can be attributed to timing inconsistency artifacts, as mentioned earlier. Figure S4 illustrates the course of frequency‐resolved SWS over the normalized R–R interval in the LV.

Table 1 provides a summary of all frequency‐resolved and composite ICCs, SWSs, as well as relative and absolute differences in SWS and LV diameter. The frequency‐resolved ICC scores were best during IVC (0.82) and DIA (0.69) for 70 Hz, and in OP (0.85) and ES (0.80) at 80 Hz with use of external vibration. For endogenous shear waves, relatively low ICCs at single frequency were observed with none exceeding 0.60. However, an acceptable ICC of 0.72 was observed during IVC based on frequency‐compounded SWS maps. Differences in SWS between the cardiac phases as compiled in Table 2 echoed these ICC trends, therefore, indicating that intrinsic‐activation cMRE is feasible in IVC and ES when performed in conjunction with generation of frequency‐compound SWS maps. In Table 2, red and blue boxes indicate results obtained with vibration‐based cMRE and intrinsic‐activation cMRE, respectively. Significant differences between cardiac phases were observed at 70 Hz with vibration, particularly between IVC and ES and between IVC and DIA. ES SWS and IVC SWS differed consistently across all vibration frequencies, however, without external vibration, SWS was significantly different between IVC and ES in the test, but not in the re‐test measurement.

**TABLE 1:** Frequency‐resolved group statistics with and without vibration.

		ICC				SWS test in m/s				SWS re‐test in m/s			
		IVC	OP	ES	DIA	IVC	OP	ES	DIA	IVC	OP	ES	DIA
With vibration	Comp	0.93	0.86	0.77	0.66	1.76 ± 0.17	2.06 ± 0.20	2.15 ± 0.23	1.93 ± 0.15	1.76 ± 0.17	2.06 ± 0.20	2.15 ± 0.23	1.96 ± 0.15
	70 Hz	0.82	0.51	0.74	0.69	1.67 ± 0.15	1.90 ± 0.18	2.02 ± 0.20	1.80 ± 0.18	1.66 ± 0.15	2.02 ± 0.18	2.02 ± 0.20	1.86 ± 0.18
	80 Hz	0.70	0.85	0.80	0.53	1.88 ± 0.14	2.15 ± 0.17	2.23 ± 0.23	1.96 ± 0.17	1.88 ± 0.14	2.13 ± 0.17	2.22 ± 0.23	2.01 ± 0.17
	90 Hz	0.72	0.43	0.46	0.37	2.14 ± 0.20	2.45 ± 0.22	2.54 ± 0.23	2.32 ± 0.21	2.07 ± 0.20	2.32 ± 0.22	2.49 ± 0.23	2.32 ± 0.21
Without vibration	Comp	0.72	0.21	0.59	0.45	1.80 ± 0.24	1.72 ± 0.17	2.00 ± 0.30	1.85 ± 0.26	1.81 ± 0.24	1.92 ± 0.17	2.16 ± 0.30	1.96 ± 0.26
	70 Hz	0.39	−0.17	0.00	0.60	1.64 ± 0.17	1.57 ± 0.14	1.83 ± 0.25	1.75 ± 0.17	1.70 ± 0.17	1.71 ± 0.14	1.92 ± 0.25	1.78 ± 0.17
	80 Hz	0.43	0.31	0.38	0.12	1.86 ± 0.20	1.84 ± 0.20	2.10 ± 0.34	1.98 ± 0.30	1.87 ± 0.20	2.02 ± 0.20	2.29 ± 0.34	2.15 ± 0.30
	90 Hz	0.26	−0.12	0.53	0.23	2.09 ± 0.34	2.12 ± 0.27	2.36 ± 0.35	2.22 ± 0.23	2.16 ± 0.34	2.26 ± 0.27	2.53 ± 0.35	2.22 ± 0.23

		Absolute difference of SWS in m/s			Relative difference of SWS in %			Absolute difference of LV diameter in %			Relative difference of LV diameter in %		
		ES‐DIA	ES‐IVC	DIA‐IVC	(ES‐DIA)/ DIA	(ES‐IVC)/ IVC	(DIA‐IVC)/ IVC	ES‐DIA	ES‐IVC	DIA‐IVC	(ES‐DIA)/ DIA	(ES‐IVC)/ IVC	(DIA‐IVC)/ IVC
With vibration	Comp	0.20 ± 0.19	0.39 ± 0.18	0.18 ± 0.13	0.10 ± 0.10	0.22 ± 0.11	0.10 ± 0.08	−8.07 ± 3.39	−10.40 ± 3.22	−2.33 ± 2.39	−8.23 ± 3.38	−10.41 ± 3.21	−2.33 ± 2.41
	70 Hz	0.19 ± 0.22	0.35 ± 0.19	0.16 ± 0.13	0.10 ± 0.12	0.21 ± 0.12	0.10 ± 0.08	−8.07 ± 3.39	−10.40 ± 3.22	−2.33 ± 2.39	−8.23 ± 3.38	−10.41 ± 3.21	−2.33 ± 2.41
	80 Hz	0.24 ± 0.24	0.35 ± 0.24	0.11 ± 0.17	0.12 ± 0.12	0.19 ± 0.13	0.06 ± 0.09	−8.07 ± 3.39	−10.40 ± 3.22	−2.33 ± 2.39	−8.23 ± 3.38	−10.41 ± 3.21	−2.33 ± 2.41
	90 Hz	0.20 ± 0.24	0.41 ± 0.24	0.21 ± 0.22	0.09 ± 0.10	0.20 ± 0.13	0.10 ± 0.11	−8.07 ± 3.39	−10.40 ± 3.22	−2.33 ± 2.39	−8.23 ± 3.38	−10.41 ± 3.21	−2.33 ± 2.41
Without vibration	Comp	0.18 ± 0.33	0.28 ± 0.34	0.10 ± 0.22	0.09 ± 0.16	0.15 ± 0.19	0.05 ± 0.12	−8.07 ± 3.39	−10.40 ± 3.22	−2.33 ± 2.39	−8.23 ± 3.38	−10.41 ± 3.21	−2.33 ± 2.41
	70 Hz	0.11 ± 0.34	0.21 ± 0.31	0.09 ± 0.20	0.06 ± 0.18	0.12 ± 0.18	0.05 ± 0.12	−8.07 ± 3.39	−10.40 ± 3.22	−2.33 ± 2.39	−8.23 ± 3.38	−10.41 ± 3.21	−2.33 ± 2.41
	80 Hz	0.13 ± 0.40	0.33 ± 0.31	0.20 ± 0.31	0.06 ± 0.20	0.18 ± 0.16	0.11 ± 0.17	−8.07 ± 3.39	−10.40 ± 3.22	−2.33 ± 2.39	−8.23 ± 3.38	−10.41 ± 3.21	−2.33 ± 2.41
	90 Hz	0.23 ± 0.38	0.32 ± 0.44	0.10 ± 0.32	0.10 ± 0.17	0.15 ± 0.21	0.05 ± 0.15	−8.07 ± 3.39	−10.40 ± 3.22	−2.33 ± 2.39	−8.23 ± 3.38	−10.41 ± 3.21	−2.33 ± 2.41

*Note*: SWS values during cardiac phases (IVC, OP, SYS, and DIA) of test and re‐test measurements and corresponding ICC scores. Values for SWS and LV diameter are tabulated as averages ± standard deviation over test and re‐test measurements.

Abbreviations: DIA, diastole; ES, end‐systole; ICC, intraclass correlation coefficient; IVC, isovolumetric contraction; LV, left ventricle; OP, optimal phase; SWS, shear wave speed.

**TABLE 2:** *p*‐Values (Bonferroni‐corrected) of SWS changes between cardiac phases IVC, ES, and DIA with vibration (blue) and without vibration (red)

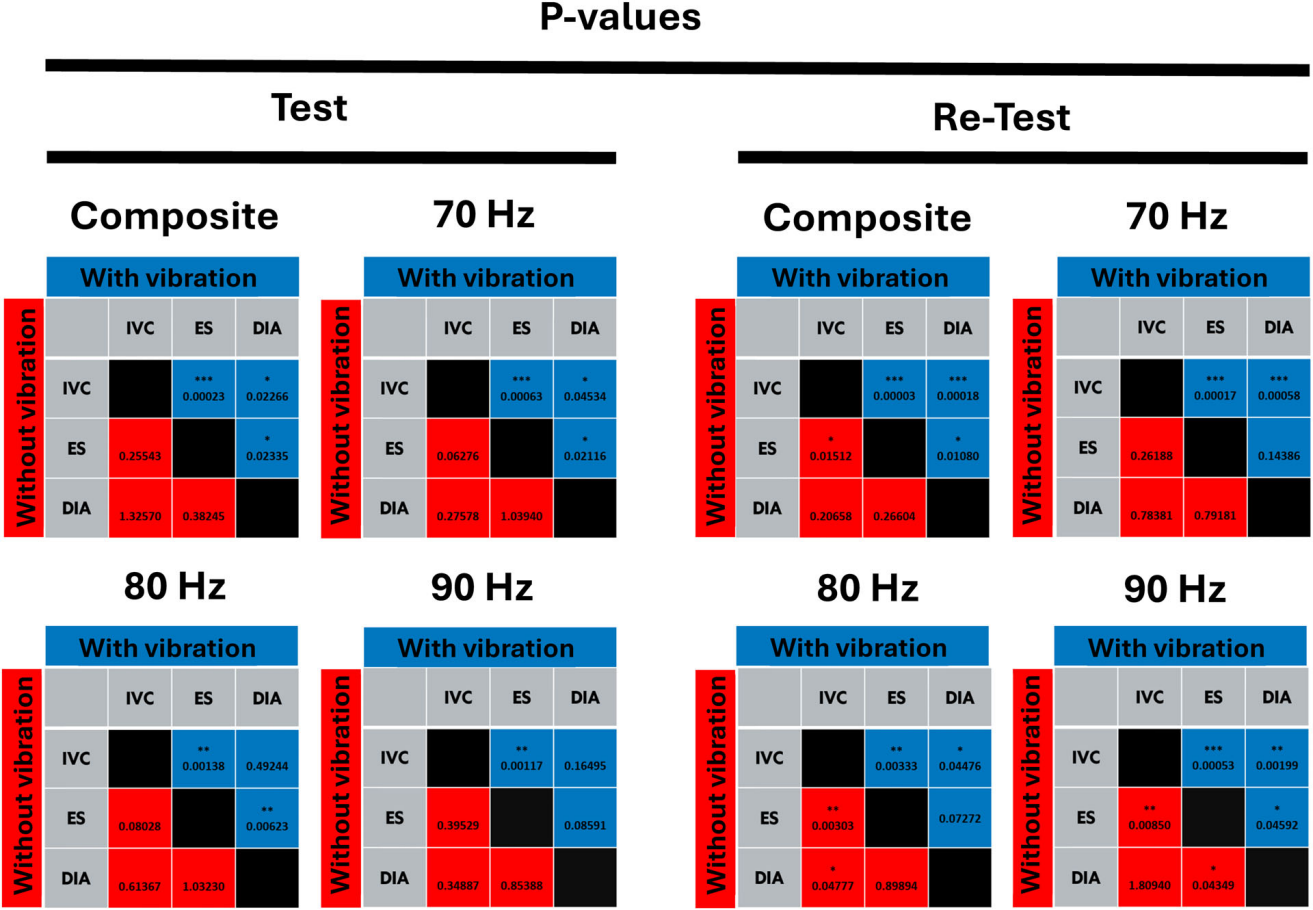			

Abbreviations: DIA, diastole; ES, end‐systole; IVC, isovolumetric contraction; OP, optimal phase.

* *p* < 0.01667.

** *p* < 0.0033.

*** *p* < 0.00033.

## 4 DISCUSSION AND CONCLUSIONS

In this study, we used high‐frame rate MRE to investigate dynamic stiffness changes during the cardiac cycle in healthy volunteers. Previous studies have shown that in vivo myocardial stiffness increases from IVC to ES and then decreases during subsequent relaxation in DIA.^12^, ^39^, ^40^, ^41^, ^42^ Although these studies used lower frame rates than our method, the observed effects of a 20% increase in peak stiffness compared with IVC and DIA were similar to our findings.^39^ However, our high frame rate of 40 Hz did not capture the steep rate of change in stiffness during IVC and after ES that one would expect from intraventricular pressure dynamics and is typically seen with invasive catheter measurement or in vivo wave amplitude‐based MRE.^12^, ^43^, ^44^ Instead, the lower rate of stiffness changes, which has also been reported for various quantitative cardiac elastography techniques, including MRE and ultrasound elastography, suggests that stiffness changes in the myocardium do not directly reflect LV pressure, but are inversely correlated with LV diameter.^5^, ^39^, ^42^ This inverse correlation (with external vibration: *R* = −0.87, *p* < 10^−6^, without external vibration: *R* = −0.54, *p* = 0.015) suggests a geometry‐related increase in stiffness, such as that caused by a nonlinear stress–strain relationship in myocardial tissue under large deformation.^45^, ^46^

To test the reproducibility of our method, we analyzed multifrequency test–re‐test ICCs with and without vibration to evaluate the consistency of cardiac MRE. Surprisingly, our data reveal that, during IVC and ES, the heart generates shear waves with amplitudes covering the entire LV and exceeding the confidence threshold of 3.6 μm (Figure S2),^38^ which raises the prospect of intrinsic cMRE in these cardiac phases. Endogenous shear wave elastography has been proposed based on pencil beam detection of septal deflection velocity^9^, ^17^ or using high‐frame rate ultrasound.^5^, ^47^ Our examinations performed without external wave stimulation offer a new way of using endogenous shear waves in MRE. The proposed frequency range was chosen for technical reasons such as ECG stability^13^ as well as maximized encoding efficiency for an effective PNR of myocardial tissue as shown in Figure 3. Here, fractional encoding based on a relatively short (8.75 ms) and first‐order nulled MEG helps to suppress dominating low‐frequency components of intrinsic heart motion by shifting the sensitivity range of cMRE toward 70 to 90 Hz. Further studies are needed to optimize the frequency sensitivity of cardiac MRE to shear waves with sufficient amplitude and wave number generated intrinsically by the heart. Our preliminary analysis showed that single‐frequency MRE performed best at 80 Hz external stimulation frequency, requiring an ICC of ≥0.7 (with optimal performance during OP), whereas the same criterion was met for endogenous shear wave MRE only during IVC when averaging over three frequencies was performed. Consequently, it remains to be tested in patients whether a shorter protocol with use of a single external vibration frequency surpasses the diagnostic performance of repeated driver‐free MRE acquisition.

It is a limitation of this study that we analyzed the reproducibility of cMRE without sensitivity analysis to cardiac pathology. However, the design of the study as a technical development focusing on physiological effects in healthy volunteers was not IRB approved for the investigation of diagnostic accuracy in patients. A technical limitation of the current method is that it requires an extended breathhold time of 23 s. However, this issue might be resolved by implementing a breathing navigator that adjusts either the slice position or the acquisition time window according to diaphragm position. The use of respiratory navigators in abdominal MRE has been demonstrated for 3D spin‐echo MRE,^48^ however, with the disadvantage of being accurate only for the first slice in the acquisition block. Respiratory navigators would also allow a greater proportion of the R–R interval to be covered. Currently, some measurements cover up to 90% of the interval, whereas others cover only 58%, so our analysis was restricted to phases where complete data were available for all subjects. Of note, our implementation of cardiac MRE is based on 2D slice acquisition, which does not account for waves traveling through the image plane. Because we encoded three space diagonals of the shear wave field, the 2D curl operator, that is, the third curl component, was readily attainable from our data after restoring orthogonality. Therefore, the consistency of wave amplitudes could be tested after Helmholtz‐Hodge decomposition based on the in‐plane curl component. This analysis, which is provided as Figure S1, showed a similar trend of wave amplitudes as seen in Figure 4. Nevertheless, it should be noted that finite‐difference curl operators are numerically limited in bounded media, discretized wave fields, and scenarios where elastic materials exhibit anisotropic properties as in myocardial tissue.^8^ This encourages alternative acquisition and postprocessing strategies such as proposed here. Overall, current cardiac MRE technology is still in the process of optimization toward better image quality, faster acquisition with more efficient motion‐encoding, and consistent quantification of mechanical tissue parameters. In this context, our study adds important novel information to the quest for mechanical imaging in cardiac disease.

In summary, we introduced high‐frame rate spiral gradient‐echo MRE to study multifrequency shear wave propagation through the human heart stimulated by either external harmonic vibration or intrinsic cardiac motion. Using external vibration, we obtained consistent SWS maps at 80 Hz during the cardiac phases from IVC to ES, including an optimal phase where relative amplitudes of externally induced shear waves were highest. In contrast, driver‐free cardiac MRE showed sufficient reproducibility only during IVC and in conjunction with multifrequency composite SWS mapping. Taken together, our study investigated the consistency of harmonic shear wave encoding in the human heart by MRE and offers a way for translation into clinical cardiac MRE.

## Funding information

German Research Foundation, Grant/Award Numbers: RTG2260 BIOQIC, CRC1340 Matrix‐in‐vision, FOR5628, 460333672 CRC1540 EBM, 540759292 Sa901/33‐1 M5.

## Supporting information


**Figure S1.** Strain and corresponding 95% confidence interval calculated based on curl field in the left ventricle (LV) with vibration and endogenous shear waves and in the liver with vibration. Strain in the LV myocardium with vibration (purple line) increased from IVC at 5.0e‐3 ± 2.0e‐3, reached the maximum of 7.0e‐3 ± 3.4e‐3 prior to OP, and decreased to its minimum 3.2e‐3 ± 1.0e‐3 before ES, and remained stable thereafter. Without vibration (red line), strain remained stable 2.0e‐3 ± 0.6e‐3 with a variability of 0.5e‐3 throughout the R–R interval. In the liver, strain remained constant with vibration (blue line) at approximately 2.0e‐3 ± 1.2e‐3, with minimal variation throughout the R–R interval.
**Figure S2.** Regions of confidence for SWS calculation using external vibration and endogenous shear waves. The regions of confidence (delineated in yellow) were identified based on MR signal intensity and a confidence threshold of 3.6 μm displacement (as proposed in Anders et al.^38^) in at least 50% of the measured encoded MEG directions of all frequencies. Both LV and the liver met the criteria for vibration, while only LV met the criteria for endogenous shear waves. DIA, diastole; ES, end‐systole; IVC, isovolumetric contraction; OP, optimal phase.
**Figure S3.** SWS maps for distinct cardiac phases based on multifrequency inversion and obtained at single frequencies. (A) With vibration. (B) Without vibration. Quality of SWS maps in diastole (DIA) is more severely degraded by accumulated cardiac timing inconsistencies between ECG and k‐space acquisition than preceding cardiac phases (IVC, OP, ES).
**Figure S4.** Time evolution of frequency‐resolved group mean SWS in the left ventricle over normalized R–R interval for test (blue) and re‐test (red) measurement. 95% confidence intervals are shown as colored shaded areas while cardiac intervals of interest are demarcated in gray. (A), (C), (E) SWS obtained with external vibration for 70, 80, and 90 Hz, respectively. (B), (D), (F) Shear wave speed (SWS) obtained from endogenous shear waves without external vibration for 70, 80, and 90 Hz, respectively. Test–re‐test reproducibility across the R–R interval was better with external vibration compared with endogenous shear waves for each frequency measured, as indicated by greater variability and lower overlap of SWS values. DIA, diastole; ES, end‐systole; IVC, isovolumetric contraction; OP, optimal phase.

**Video S1.** The effect of SVD denoising on raw phase images with and without vibration. Representative wave phases acquired at 80 Hz sampling are shown to illustrate the effect of SVD denoising, with the left ventricular myocardium delineated in yellow. Group mean signal differences within the myocardium are negligible, at 0.00 ± 0.89 rad with vibration and 0.00 ± 1.01 rad without vibration, confirming that SVD does not induce spurious wave motion.

**Video S2.** Displacement fields acquired during the optimal phase in a representative volunteer. The animation shows the propagation of shear waves for each frequency (70, 80, and 90 Hz) for the three motion‐encoding directions (+x + y + z, +x −y + z, +x + y −z) with vibration and endogenous shear waves. ROIs were defined by anatomical structures visible in the MRE magnitude images, in which at least 50% of the MEG direction frequency pairs surpass the deflection threshold of 3.6 μm. Deflection was highest at 70 Hz and lowest at 90 Hz.

## APPENDIX A

### A.1 SVD denoising in cardiac MRE

This appendix briefly introduces the concept of SVD denoising in cardiac MRE. cMRE typically suffers from encoding a relatively high level of phase signal associated with rapid wall motion, blood turbulence, and inconsistent respiratory pauses. This unwanted signal is superimposed to the desired, sinusoidal oscillatory phase. While the wanted phase signal is expected to propagate in coherent spatiotemporal patterns, the incoherent, unwanted, signal is stochastically distributed in space and time. Therefore, spatiotemporal filters such as those introduced by Manduca et al.^49^ can effectively separate propagating shear waves from stochastic phase fluctuations. A similar concept is SVD denoising based on MRE images converted to 2D space–time arrays. However, the temporal support in the time domain is typically very low (4–8 time steps over a wave phase) compared to the image domain (number of pixels), making SVD applied to a spatiotemporal ensemble in MRE unbalanced and therefore ineffective.^50^ In contrast, our cMRE sequence favors SVD denoising because the spatiotemporal ensemble is better balanced with image domain sizes from 54 × 40 to 62 × 54 (pixels within the cropped heart‐region) and time domain sizes from 25 × 6 to 35 × 6 (cardiac phases times wave dynamics). This situation applies to individual series of single‐component, single‐frequency wave images and resembles the recommended denoising for cardiac ultrasound elastography.^27^, ^51^

SVD basically transforms a rectangular matrix into an orthonormal basis. Therefore, the MRE acquired spatiotemporal 3D array $A\in {\mathbb{C}}^{n_{x}\times n_{y}\times n_{t}}$ is converted to a 2D matrix $B\in {\mathbb{C}}^{n_{x}n_{y}\times n_{t}}$ (with n denoting the numbers of pixels along the spatial x−y or temporal t‐domains), which can be defined as:

(A1)
$$B=U\sum V^{T}.$$

$U\in {\mathbb{C}}^{n_{x}n_{y}\times n_{x}n_{y}}$ is the spatial eigenvector, $V^{T}\in {\mathbb{C}}^{n_{t}\times n_{t}}$ is the transposed temporal eigenvector, and $\sum \in {\mathbb{C}}^{n_{x}n_{y}\times n_{t}}$ contains the eigenvalues $\sigma_{i}$ in descending order. While higher eigenvalues correspond to coherent cardiac motion and propagating shear waves, lower eigenvalues are considered noise. Therefore, ∑ can be used to generate a filtered spatiotemporal matrix $B_{F}$ by limiting the number of considered eigenvalues *R* to retain only main signal contributions

(A2)
$$\sum_{F}=\text{diag}\left(\sigma_{1},\sigma_{2},\ldots ,\sigma_{R},0,0,\ldots ,0\right).$$

$\sum_{F}$ is used for back‐transformation according to Equation (A1) yielding $B_{F}$, which represents a low‐rank approximation of B with preserved coherent motion of myocardial geometry and shear waves while suppressed incoherent noise. In this study, the eigenvalue threshold was empirically set to preserve at least 97% of the total variance corresponding to the bins 1 to 20, as discussed in Jolliffe et al.^52^
