# Supplementary material for: Time‐resolved mapping of myocardial stiffness using 2D multifrequency spiral MR elastography with and without external vibration
Source: Magn Reson Med. 2025 Jul 28;94(6):2421–34. doi: 10.1002/mrm.70007 (PMC12501707; doi:10.1002/mrm.70007)
Supplement: Supplementary file 1 — Figure S1. Strain and corresponding 95% confidence interval calculated based on curl field in the left ventricle (LV) with vibration and endogenous shear waves and in the liver with vibration. Strain in the LV myocardium with vibration (purple line) increased from IVC at 5.0e‐3 ± 2.0e‐3, reached the maximum of 7.0e‐3 ± 3.4e‐3 prior to OP, and decreased to its minimum 3.2e‐3 ± 1.0e‐3 before ES, and remained stable thereafter. Without vibration (red line), strain remained stable 2.0e‐3 ± 0.6e‐3 with a variability of 0.5e‐3 throughout the R–R interval. In the liver, strain remained constant with vibration (blue line) at approximately 2.0e‐3 ± 1.2e‐3, with minimal variation throughout the R–R interval. Figure S2. Regions of confidence for SWS calculation using external vibration and endogenous shear waves. The regions of confidence (delineated in yellow) were identified based on MR signal intensity and a confidence threshold of 3.6 μm displacement (as proposed in Anders et al. 38 ) in at least 50% of the measured encoded MEG directions of all frequencies. Both LV and the liver met the criteria for vibration, while only LV met the criteria for endogenous shear waves. DIA, diastole; ES, end‐systole; IVC, isovolumetric contraction; OP, optimal phase. Figure S3. SWS maps for distinct cardiac phases based on multifrequency inversion and obtained at single frequencies. (A) With vibration. (B) Without vibration. Quality of SWS maps in diastole (DIA) is more severely degraded by accumulated cardiac timing inconsistencies between ECG and k‐space acquisition than preceding cardiac phases (IVC, OP, ES). Figure S4. Time evolution of frequency‐resolved group mean SWS in the left ventricle over normalized R–R interval for test (blue) and re‐test (red) measurement. 95% confidence intervals are shown as colored shaded areas while cardiac intervals of interest are demarcated in gray. (A), (C), (E) SWS obtained with external vibration for 70, 80, and 90 Hz, respectively. (B), (D), (F) Sh [file MRM-94-2421-s001.docx]

Supporting information on

**Time-resolved mapping of myocardial stiffness using two-dimensional multifrequency spiral magnetic resonance elastography with and without external vibration**

Matthias S. Anders^1^, Carsten Warmuth^2^, Tom Meyer^2^, Helge Herthum^3^, Mehrgan Shahryari^2^, Jakob Schattenfroh^2^, Corona Metz^1^, Jan Bieling^2^, Josef Pfeuffer^4^, Simon Veldhoen^1^, Jeanette Schulz-Menger^5,6,7,8^, Tobias Schaeffter^9,10^, Jing Guo^2^, Heiko Tzschaetzsch^11^, Ingolf Sack^2^

**Affiliations** *^1^Division of Pediatric Radiology,* *Charité – Universitätsmedizin Berlin, Corporate Member of Freie Universität Berlin and Humboldt-Universität zu Berlin, Berlin, Germany*

*^2^Department of Radiology,* *Charité – Universitätsmedizin Berlin, Corporate Member of Freie Universität Berlin and Humboldt-Universität zu Berlin, Berlin, Germany*

*^3^Berlin Center for Advanced Neuroimaging (BCAN), Berlin, Germany, Corporate Member of Freie Universität Berlin, Berlin Institute of Health and Humboldt-Universität zu Berlin, Berlin, Germany*

*^4^Application Development, Siemens Healthineers AG, Erlangen, Germany*

*^5^Charité – Universitätsmedizin Berlin, Corporate Member of Freie Universität Berlin and Humboldt-Universität zu Berlin, Berlin, Germany*

*^6^Working Group On CMR, Experimental and Clinical Research Center, a cooperation between Charité - Universitätsmedizin Berlin and Max Delbrück Center for Molecular Medicine in the Helmholtz Association, Berlin, Germany*

*^7^DZHK (German Center for Cardiovascular Research), partner site Berlin, Berlin, Germany*

*^8^Department of Cardiology and Nephrology, HELIOS Hospital Berlin-Buch, Berlin, Germany*

*^9^Physikalisch-Technische Bundesanstalt (PTB), Braunschweig and Berlin, Berlin, Germany*

*^10^Department of Medical Engineering, Technische Universität Berlin, Einstein Center Digital Future, Germany*

*^11^Institute of Medical Informatics, Charité – Universitätsmedizin Berlin, Corporate Member of Freie Universität Berlin and Humboldt-Universität zu Berlin, Berlin, Germany*


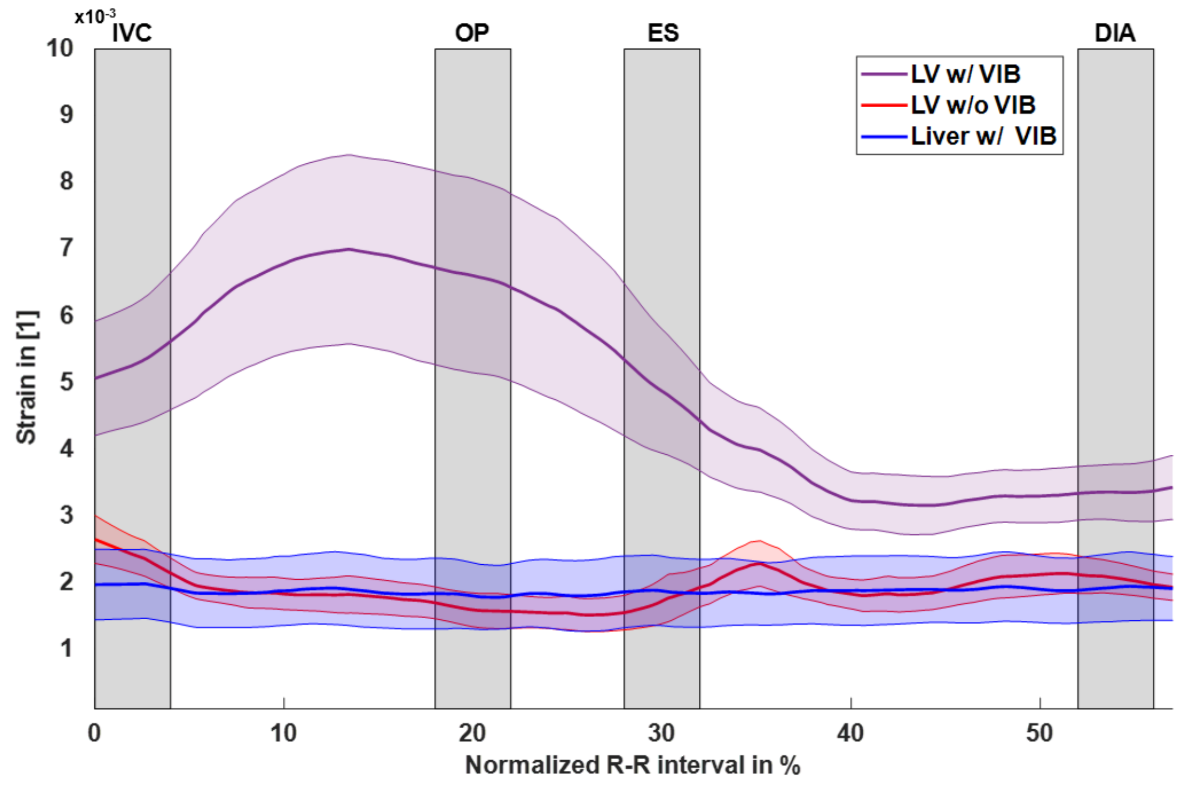


**Figure S1:** Strain and corresponding 95% confidence interval calculated based on curl field in the left ventricle (LV) with vibration and endogenous shear waves and in the liver with vibration. Strain in the LV myocardium with vibration (purple line) increased from IVC at 5.0e-3±2.0e-3, reached the maximum of 7.0e-3±3.4e-3 prior to OP, and decreased to its minimum 3.2e-3±1.0e-3 before ES, and remained stable thereafter. Without vibration (red line), strain remained stable 2.0e-3±0.6e-3 with a variability of 0.5e-3 throughout the R-R interval. In the liver, strain remained constant with vibration (blue line) at approximately 2.0e-3±1.2e-3, with minimal variation throughout the R-R interval.


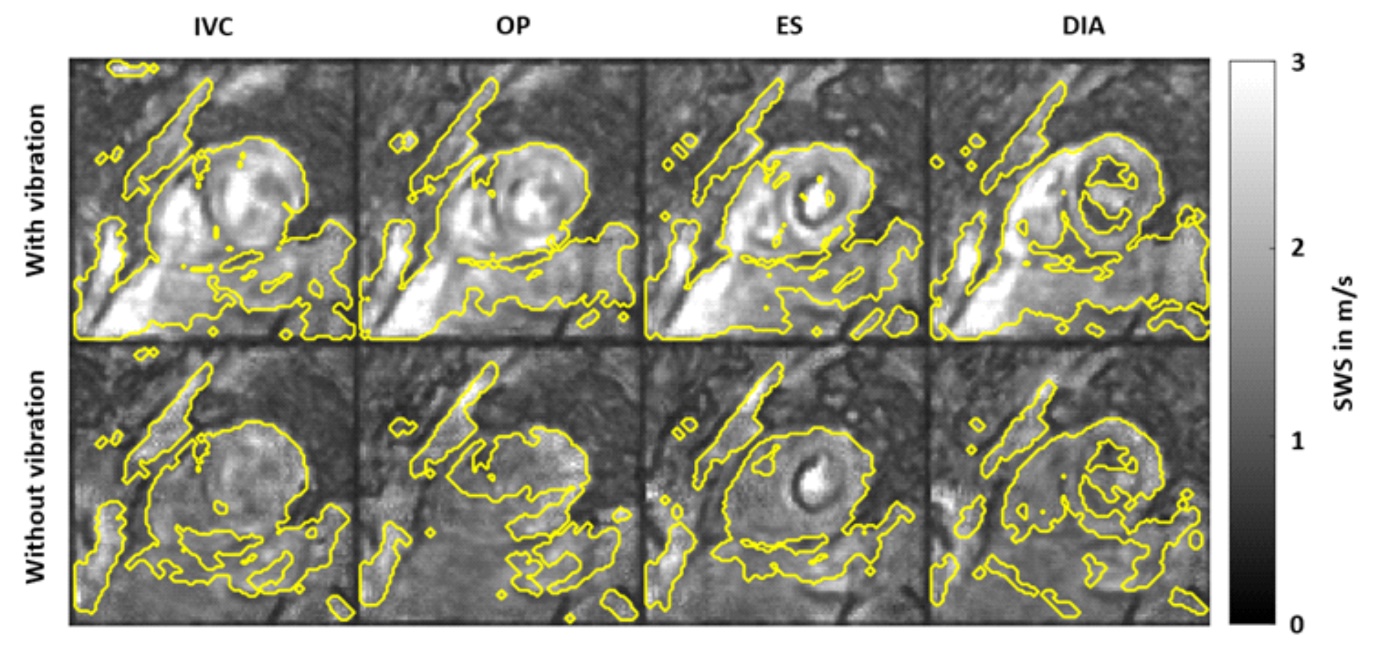


**Figure S2:** Regions of confidence for SWS calculation using external vibration and endogenous shear waves. The regions of confidence (delineated in yellow) were identified based on MR signal intensity and a confidence threshold of 3.6 µm displacement (as proposed in Anders et al.^38^) in at least 50% of the measured encoded MEG directions of all frequencies. Both LV and the liver met the criteria for vibration, while only LV met the criteria for endogenous shear waves. IVC: isovolumetric contraction, OP: optimal phase, ES: end-systole, DIA: diastole


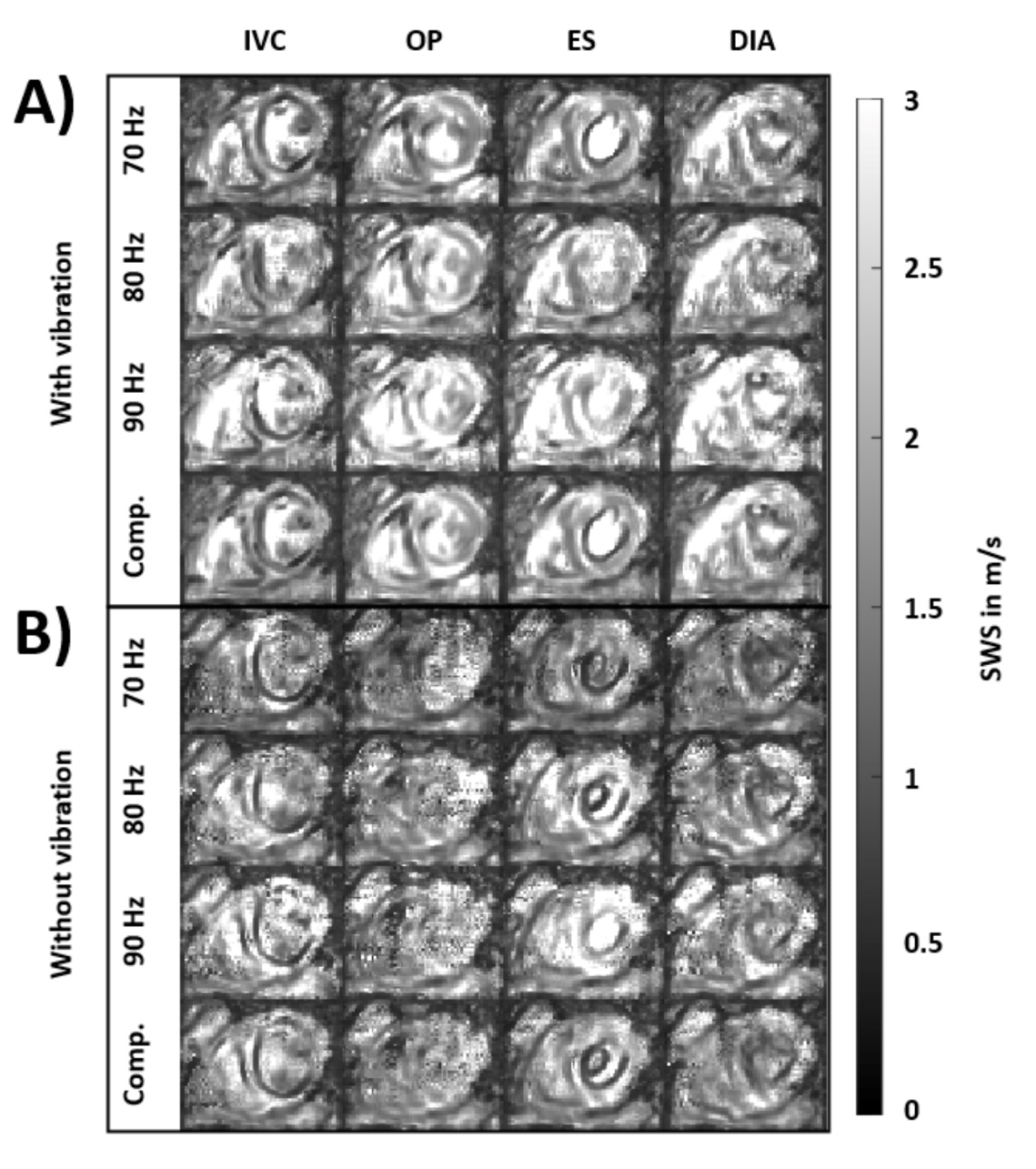


**Figure S3:** SWS maps for distinct cardiac phases based on multifrequency inversion and obtained at single frequencies. **(A)** With vibration. **(B)** Without vibration. Quality of SWS maps in diastole (DIA) is more severely degraded by accumulated cardiac timing inconsistencies between ECG and k-space acquisition than preceding cardiac phases (IVC, OP, ES).


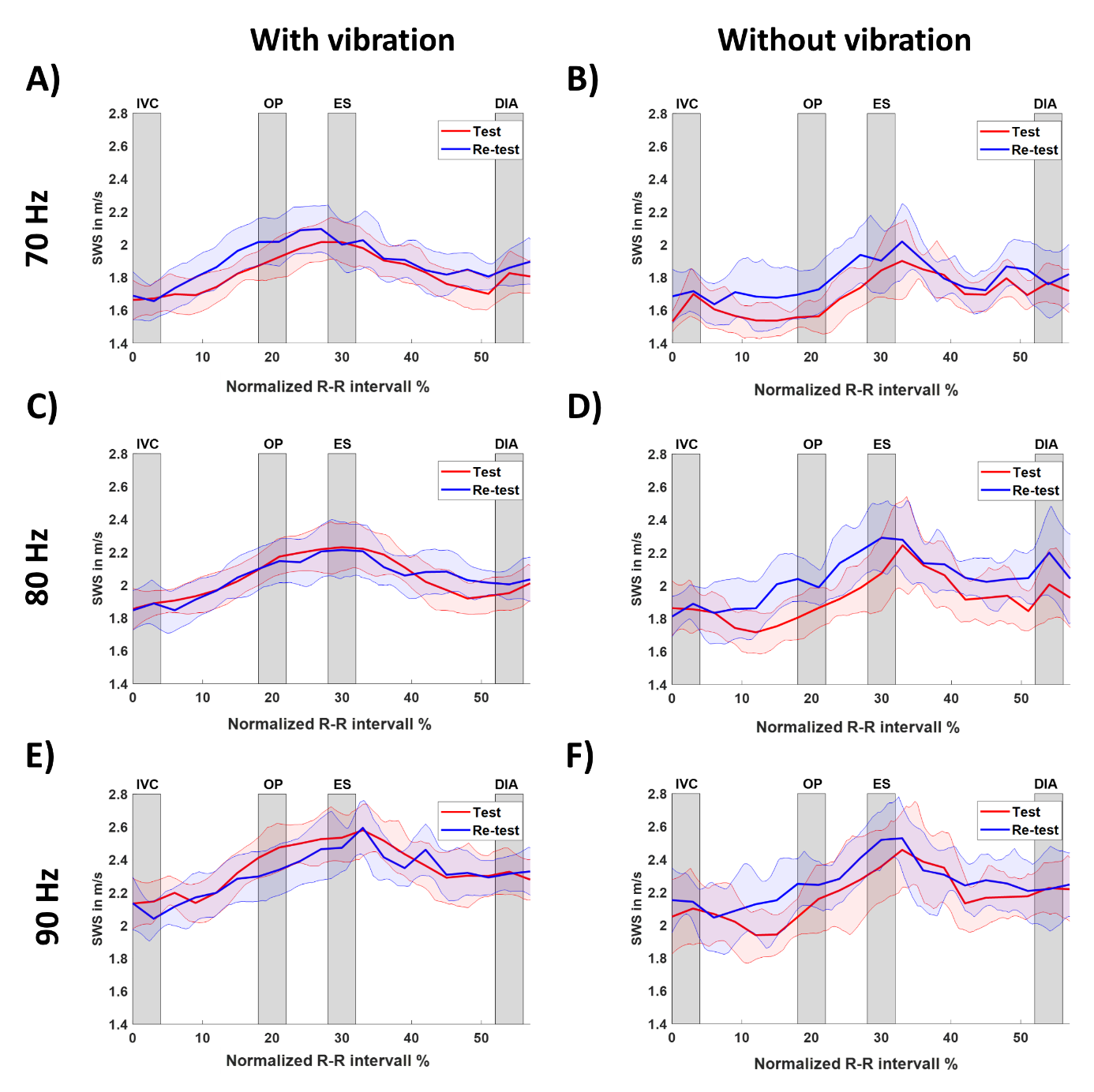


**Figure S4:** Time evolution of frequency-resolved group mean SWS in the left ventricle over normalized R-R interval for test (blue) and re-test (red) measurement. 95% confidence intervals are shown as colored shaded areas while cardiac intervals of interest are demarcated in gray. **(A)**, **(C)**, **(E)** SWS obtained with external vibration for 70, 80, and 90 Hz, respectively. **(B)**, **(D)**, **(F)** SWS obtained from endogenous shear waves without external vibration for 70 Hz, 80 Hz, and 90 Hz, respectively. Test–re-test reproducibility across the R-R interval was better with external vibration compared with endogenous shear waves for each frequency measured, as indicated by greater variability and lower overlap of SWS values. IVC: isovolumetric contraction, OP: optimal phase, ES: end-systole, DIA: diastole.
